# Time to Rectify the Neglect? Audit on Prescription Writing the Neglected skill. Audit Ref No: AU/006/01/2021

**DOI:** 10.1192/bjo.2022.437

**Published:** 2022-06-20

**Authors:** Amit Fulmali, Sara Sheik, Amir-Humza Suleman, Faryal Rana, Ruth Bloxam, Lubna Abdallah, Ranjit Mahanta

**Affiliations:** Surrey and Borders Partnership NHS Foundation Trust, Guildford, United Kingdom

## Abstract

**Aims:**

Prescribing is a neglected skill amongst trainees. Prescription errors can harm patients. A recent Economic analysis published in BMJ Quality & Safety. Estimated that 237 million medication errors occur in England annually. Costing the NHS £98,462,582. Prescribing errors contributed to 21% of the total errors. It is important that all prescribers are aware of principles of safe prescribing. Our aim is to is to establish whether our practice is meeting standards of prescription writing in old age psychiatry ward setup.

**Methods:**

We used prescription standards set by BMA, BNF and SABP (Surrey and Borders Partnership Foundation NHS Trust) to assess all prescriptions. The following parameters were checked: GMC number, Sign, Name of Doctor, Name of drug, Indication, Dose, Route, Frequency, Original start date, current Date, medication timings.

**Data collection and handling:**

We performed a closed loop audit. A retrospective data of 228 prescriptions were collected from August 2020 to January 2021 from patients admitted in Victoria Ward. The data were analysed and presented at departmental meeting. Re-training on prescription writing conducted. New data was prospectively collected comprising of 230 prescriptions from March 2021 to June 2021 to complete the audit cycle.

Excel sheet was used to collect the data and to get the results. All Prescription charts were collected from SystmOne (clinical software system). Data from both the Audit's were analysed and compared.

**Results:**

We found errors in all parameters, except for medication timings. Comparison of the data from the first audit and re-audit showed an increase in prescription errors.

There was an increased 20.33% error in writing GMC number, 16.87% error in writing name of the doctor, 12.94% error in indication and 5% error in original start date. There was improvement of 10.88% in one parameter, “Name of the drug”.

**Conclusion:**

A significant error was found in writing the GMC number and the Doctor's name, despite regular training during induction. There are no clear guidelines on the writing of GMC registration being compulsory on Drug chart. With one exception if online and you are not the patient's regular prescriber, then your GMC registration number is required.

**Recommendations:**

1. We recommended the trust to issue stamps with GMC number and doctor's name.
2. Re-audit in 6 months’ time after introduction of the stamps.
3. Quarterly regular training of new Trainee doctors.

**Service improvements:**

After the Audit was submitted locally, stamps were introduced and issued to junior doctors at Victoria Ward by the Trust.

